# Diabetes Causes Dysfunctional Dopamine Neurotransmission Favoring Nigrostriatal Degeneration in Mice

**DOI:** 10.1002/mds.28124

**Published:** 2020-07-15

**Authors:** Iara Pérez‐Taboada, Samuel Alberquilla, Eduardo D. Martín, Rishi Anand, Stefania Vietti‐Michelina, Nchimunya N. Tebeka, James Cantley, Stephanie J. Cragg, Rosario Moratalla, Mario Vallejo

**Affiliations:** ^1^ Instituto de Investigaciones Biomédicas Alberto Sols, Consejo Superior de Investigaciones Científicas (CSIC)/Universidad Autónoma de Madrid Madrid Spain; ^2^ Centro de Investigación Biomédica en Red de Diabetes y Enfermedades Metabólicas Asociadas CIBERDEM Madrid Spain; ^3^ Instituto Cajal, Consejo Superior de Investigaciones Científicas (CSIC) Madrid Spain; ^4^ Department of Physiology, Anatomy and Genetics University of Oxford Oxford United Kingdom; ^5^ Division of Systems Medicine University of Dundee, Ninewells Hospital & Medical School Dundee United Kingdom; ^6^ Oxford Parkinson's Disease Centre University of Oxford Oxford United Kingdom; ^7^ CIBERNED, Instituto de Salud Carlos III Madrid Spain

**Keywords:** hyperglycemia, oxidative stress, nigrostriatal neurons, dopamine, presynaptic proteins

## Abstract

**Background:**

Numerous studies indicate an association between neurodegenerative and metabolic diseases. Although still a matter of debate, growing evidence from epidemiological and animal studies indicate that preexisting diabetes increases the risk to develop Parkinson's disease. However, the mechanisms of such an association are unknown.

**Objectives:**

We investigated whether diabetes alters striatal dopamine neurotransmission and assessed the vulnerability of nigrostriatal neurons to neurodegeneration.

**Methods:**

We used streptozotocin‐treated and genetically diabetic *db/db* mice. Expression of oxidative stress and nigrostriatal neuronal markers and levels of dopamine and its metabolites were monitored. Dopamine release and uptake were assessed using fast‐scan cyclic voltammetry. 6‐Hydroxydopamine was unilaterally injected into the striatum using stereotaxic surgery. Motor performance was scored using specific tests.

**Results:**

Diabetes resulted in oxidative stress and decreased levels of dopamine and its metabolites in the striatum. Levels of proteins regulating dopamine release and uptake, including the dopamine transporter, the Girk2 potassium channel, the vesicular monoamine transporter 2, and the presynaptic vesicle protein synaptobrevin‐2, were decreased in diabetic mice. Electrically evoked levels of extracellular dopamine in the striatum were enhanced, and altered dopamine uptake was observed. Striatal microinjections of a subthreshold dose of the neurotoxin 6‐hydroxydopamine in diabetic mice, insufficient to cause motor alterations in nondiabetic animals, resulted in motor impairment, higher loss of striatal dopaminergic axons, and decreased neuronal cell bodies in the substantia nigra.

**Conclusions:**

Our results indicate that diabetes promotes striatal oxidative stress, alters dopamine neurotransmission, and increases vulnerability to neurodegenerative damage leading to motor impairment. © 2020 The Authors. *Movement Disorders* published by Wiley Periodicals LLC on behalf of International Parkinson and Movement Disorder Society.

Metabolic and neurodegenerative disorders have increased their incidence worldwide for several decades. Diabetes has reached epidemic proportions, and its incidence is predicted to double by 2030 relative to figures obtained in 2000.^1^ Alzheimer's (AD) and Parkinson's diseases (PD) are becoming more prevalent in the elderly population, and the number of individuals affected by PD is predicted to increase in a similar proportion to that of diabetes.^2^ Thus, determining whether the presence of prevalent metabolic disorders increases the risk of neurodegenerative diseases is of great importance.

Evidence that diabetes constitutes a risk factor associated with increased incidence of neurodegenerative diseases has been accumulating in recent years. This association has been more clearly observed in epidemiological and experimental studies in the case of AD, in which the greater risk of cognitive decline in diabetic patients is well documented.^3^, ^4^ In the case of PD, a number of epidemiological studies indicate the existence of increased incidence in association with preexisting diabetes.^5^, ^6^, ^7^, ^8^, ^9^, ^10^, ^11^ Insulin resistance in parkinsonian patients is associated with accelerated disease progression, increased severity of motor impairment, and increased risk of PD dementia.^12^, ^13^, ^14^ Furthermore, a number of patients with diabetes mellitus without PD exhibit pathologies related to subclinical striatal dopaminergic dysfunction.^15^ Diabetes and PD share common etiopathogenic mechanisms,^16^, ^17^ and the finding of α‐synuclein inclusions in pancreatic β cells of diabetic patients further supports an association between both diseases.^18^

Despite these findings, other studies have reported that this association is weak or even nonexistent.^19^, ^20^, ^21^, ^22^ These discrepancies have been attributed to heterogeneity in self‐reporting or diagnostic criteria, differences in study size or design, genetic ancestry or lifestyle habits of patients, or presence of other poorly adjusted confounders. Therefore, from an epidemiological point of view, whether the preexistence of diabetes increases the risk of developing PD remains a matter of debate.

Although the most recent reports, including large cohort studies, support an association between preceding diabetes mellitus and PD, the possible mechanisms by which diabetes favors the development of PD are unknown. Animal and human studies indicate that early impairment of synaptic function occurs before neurodegeneration takes place.^23^, ^24^, ^25^ Therefore, we investigated whether the presence of diabetes in mice results in changes in dopaminergic neurotransmission in the striatum that could correlate with increased sensitization of nigrostriatal neurons to neurodegeneration.

## Materials and Methods

### Animals

We used C57BL/6J mice (n = 166) to generate streptozotocin (STZ)‐induced diabetes. Diabetic BKS‐D‐Lepr *db/db* mice, in which hyperglycemia develops at 4 to 8 weeks of age,^26^ or nondiabetic heterozygote BKS‐D‐Lepr *db/*+ mice (Janvier Labs, Le Genest‐Saint‐Isle, France) were also used (n = 79; body weights at the time of testing: *db/db*, 48.2 ± 0.82; *db/+*, 27.5 ± 0.75 g). All were 15‐ to 20‐week‐old males housed under a 12:12‐hour dark/light cycle at 22 ± 2°C with food and water ad libitum. The Consejo Superior de Investigaciones Científicas (CSIC) Ethics Committee or the University of Oxford Ethical Review Board approved the experimental protocols following European Union (63/2010/EU) and Spanish legislation (RD 53/2013) or the United Kingdom Animals (Scientific Procedures) Act (1986).

### STZ‐Dependent Diabetes

STZ (50 mg/kg, i.p.; Sigma‐Aldrich, St. Louis, MO) was administered for 5 consecutive days.^27^, ^28^ Mice were considered diabetic when glucose levels monitored with a glucometer (Accu Chek Performa Nano; Roche, Basel, Switzerland), using tail blood after a 4‐hour fast, were >250 mg/dL. Control mice were injected with vehicle (10 mM of sodium citrate, 0.9% NaCl; pH 4.5). When indicated, sustained‐release insulin pellets (LinShin, Toronto, Ontario, Canada) were implanted subcutaneously after STZ treatment, and stable recovery of blood glucose levels was monitored weekly (Supporting Information Figs. S1 and S2).

### Brain Dissection

Dissections of caudate putamen (CPu) and substantia nigra (SN) were performed as shown in Supporting Information Figure S3.

### Reverse Transcription Quantitative Polymerase Chain Reaction

RNA was extracted using TRI Reagent Solution (Ambion, Inc, Austin, TX) from freshly dissected small blocks of mesencephalic tissue containing the SN. SYBR Green detection (Applied Biosystems, Hercules, CA) was used, and values were normalized to glyceraldehyde 3‐phosphate dehydrogenase (*Gapdh*) mRNA levels using the double delta threshold cycle (Ct) method. Stability of *Gapdh* as a reference gene was confirmed using GeNorm^29^ (Supporting Information Fig. S4) and is in agreement with previous studies showing stable expression in diabetic mice.^28^, ^30^ Primer sequences were obtained from the MGH‐Harvard PrimerBank (https://pga.mgh.harvard.edu/primerbank).

### Western Blot

Protein extracts were prepared from the SN or the CPu freshly dissected on ice. Antibodies used are indicated in Supporting Information Table S1. Bands were visualized with ECL (GE Healthcare, Waukesha, WI), and densitometry was performed using ImageJ (http://rsbweb.nih.gov/ij/).

### Catalase Activity, Glutathione, and 4‐Hydroxynonenal

The following kits were used: catalase activity and glutathione (GSH) production, Cayman Chemical (Ann Arbor, MI); 4‐hydroxynonenal (4‐HNE) content, Cell Biolabs HNE Adduct Competitive ELISA Kit (Cell Biolabs, Inc., San Diego CA). Samples were processed from freshly dissected CPu as specified by the manufacturers.

### High‐Performance Liquid Chromatography

The CPu was rapidly removed on ice and frozen at –80°C. Monoamines and their metabolites were determined using an ESA Coulochem III detector.^31^

### Fast‐Scan Cyclic Voltammetry

Fast‐scan cyclic voltammetry (FSCV) was performed as described.^32^, ^33^ Freshly prepared coronal slices (300 μm) were incubated in artificial cerebrospinal fluid (aCSF; saturated with 95% O_2_/5% CO_2_) containing 10 mM glucose. A hemisphere containing both CPu and nucleus accumbens core (NAc) from each of a diabetic and nondiabetic mouse were placed in the recording chamber together and superfused with aCSF. Extracellular concentrations of dopamine ([DA]_o_) evoked by local electrical stimuli (200 μs, 0.6 mA) were monitored at single‐use carbon‐fiber microelectrodes (7–10 μm in diameter) fabricated in‐house (tip length: 50–100 μm) using a Millar voltammeter (Julian Millar, Barts, and the London School of Medicine and Dentistry). Evoked [DA]_o_ was sampled at three recording sites in the dorsolateral striatum (DLS) and two in the NAc. The order of location and diabetic/nondiabetic sampling was randomized for each pair of slices.

### Microinjections of 6‐OHDA

A subthreshold dose (2.5 μg/μL in two deposits of 2 μL each) of 6‐OHDA (10 mM solution in 0.9% NaCl/0.2% ascorbic acid; Sigma‐Aldrich, Madrid, Spain), defined as a dose that does not induce motor impairment in nondiabetic mice, corresponding to half the dose required to induce overt neurodegeneration of nigrostriatal axons,^34^ was injected unilaterally under 2% isoflurane anesthesia into the dorsal striatum using a Hamilton syringe. Stereotaxic coordinates were: AP = 0.65, L = 2.0, DV_1_ = –4 and DV_2_ = –3.5.^35^ In STZ‐treated mice, 6‐OHDA was injected either 2 or 4 weeks after the onset of hyperglycemia. The mortality rate was 8% in nondiabetic mice and 30% in diabetic animals, in consonance with previously reported figures.^36^ Mice were tested 10 days after 6‐OHDA administration.

### Motor Function

Mice were tested at 24‐hour intervals and killed 24 hours after the last test (15 days after 6‐OHDA administration). The following tests were used:

#### Challenging Traversal Beam Test

Mice were trained for 2 consecutive days to walk across the length of a 1‐m‐long beam with a grid surface becoming gradually narrower (from 3.5 to 0.5 cm). On the test day, mice were videotaped and the time to traverse the beam and the number of paw misses when stepping were scored.^37^

#### Cylinder Test

Mice were individually placed in a transparent cylinder to assess the asymmetry in the spontaneous use of forelimbs. The number of wall contacts made with the ipsilateral and the contralateral forepaws (relative to the 6‐OHDA injection side) during 3 minutes were videotaped and scored.^38^

#### Rota‐Rod Test

We used an accelerating rota‐rod apparatus (Hugo Basile, Gemonio, Italy) as described.^31^ Rotation accelerated from 4 to 40 rpm (or 4–20 rpm for *db/db* mice) within 5 minutes, and the time to fall was determined.

#### Amphetamine‐Induced Rotation Test

Rotational behavior was assessed only with *db/db* mice because their obesity prevented the use of the cylinder and challenging traversal beam tests. Following administration of D‐amphetamine sulfate (5 mg/kg, i.p.; Sigma‐Aldrich), full‐body turns toward the ipsilateral 6‐OHDA injection side were scored using rotometer bowls.^39^

### Immunohistochemistry and Lesion Quantification

All animals used for histological assessment had been tested for motor performance. Coronal free‐floating sections (30 μm) from a slicing vibratome were incubated overnight with tyrosine hydroxylase (TH) or dopamine transporter (DAT) antibodies (Supporting Information Table S1) for diaminobenzidine‐immunoperoxidase staining. Staining intensity was quantified by digital image analysis (Imaging Research Inc, Linton, UK).^40^

### Stereological Quantification of TH‐Positive Neurons

The number of TH‐positive neurons in the SN pars compacta (SNc) was determined on coronal mesencephalic sections using stereology on an optical fractionator.^40^

### Statistical Analyses

Data were analyzed by one‐ or two‐way analysis of variance (ANOVA), followed by Dunnett's or Bonferroni's tests, respectively, or by two‐tailed Student's *t* test, using GraphPad Prism software (GraphPad Software Inc., La Jolla, CA). Data are expressed as mean ± SEM.

## Results

**FIG. 1.**
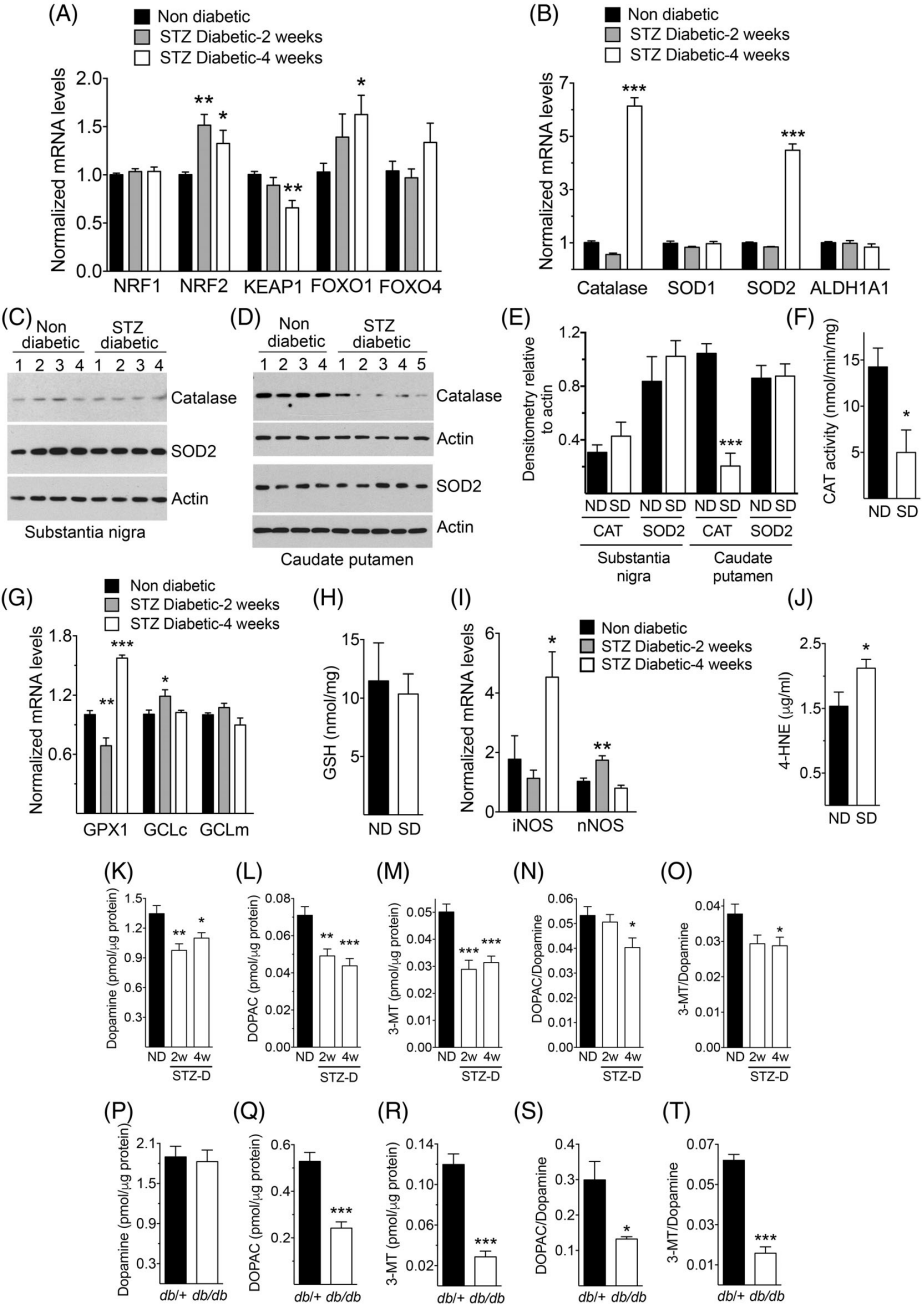
Oxidative stress in the SN and CPu. (**A,B**) Levels of mRNAs encoding oxidative stress‐related transcription factors (**A**) or oxidative stress‐scavenging enzymes (**B**) in the SN (n = 4–8 in [A] and 6–8 in [B]). (**C,D**) Western blots with lysates from the SN (**C**) or the CPu (**D**) of nondiabetic or STZ‐treated 4‐week diabetic mice. The numbers on top of each lane indicate individual mice from which samples were obtained. (**E**) Densitometric quantification of the intensities of the catalase (CAT) and SOD2 bands from panels (**C**) and (**D**). (**F**) Catalase activity in CPu homogenates (n = 3 per group). (**G**) Expression of mRNAs encoding glutathione‐related enzymes in the SN (n = 5–8 per group). (**H**) GSH production in CPu homogenates (n = 8 per group). (**I**) Expression of mRNAs encoding iNOS or nNOS in the SN (n = 6–7 per group). (**J**) Levels of the ROS indicator product 4‐HNE in CPu homogenates (n = 11 for ND and 9 for SD). (**K**–**T**) Levels of dopamine and its metabolites, DOPAC and 3‐MT, in CPu homogenates from nondiabetic and STZ‐treated diabetic mice (**K–O**, n = 6 per group) or from *db/*+ control and *db/db* diabetic mice (**P**–**T**, n = 4 per group). ^*^ *P* < 0.05; ^**^ *P* < 0.01; ^***^ *P* < 0.001 versus nondiabetic controls, one‐way ANOVA followed by Dunnett's post‐hoc test (**A,B,G,I,K–O**) or Student's *t* test (**E,F,J,Q–T**). ND, nondiabetic; SD, STZ‐treated diabetic (4‐week, unless otherwise indicated in [**K–O**]); GPX1, glutathione peroxidase 1; GCL, glutamate‐cysteine ligase, catalytic (c) or modulatory (m) subunits

To investigate whether hyperglycemia and insulin depletion are sufficient to generate oxidative stress in nigrostriatal neurons, we used STZ‐treated diabetic mice. Expression of mRNAs encoding the oxidative stress‐related transcription factors, nuclear factor erythroid 2–related factor 2 (Nrf2) or forkhead box O1 (FoxO1),^41^, ^42^ in the SN were higher than in controls, whereas expression of the mRNA encoding the Nrf2 inhibitor Keap1 was decreased (Fig. 1A). Consistently, we detected elevated expression of mRNAs encoding the oxidative stress‐scavenging enzymes, catalase and superoxide dismutase (SOD) 2, but not of those encoding SOD1 or aldehyde dehydrogenase 1a1 (Fig. 1B), suggesting the presence of a defense response against oxidative stress. Changes in SOD2 mRNA did not translate into significant differences in protein levels between STZ‐diabetic and control mice in either the SN or CPu (Fig. 1C–E). Remarkably, catalase protein levels were similar in the SN in both groups, but were significantly decreased in the CPu of 4‐week STZ‐diabetic mice (Fig. 1C–E), suggesting impaired axonal transport or enhanced degradation in striatal fibers. Consistently, STZ‐diabetic mice showed decreased catalase activity in the CPu (Fig. 1F).

Glutathione peroxidase 1 mRNA was transiently decreased in the SN in 2‐week STZ‐diabetic mice, but became elevated relative to controls after 4 weeks (Fig. 1G). The mRNA of the antioxidant gene glutamate‐cysteine ligase, catalytic subunit (GCLc), encoding the rate‐limiting enzyme for the synthesis of glutathione,^43^ was transiently elevated in diabetic mice, an effect not observed with the modifier subunit (GCLm; Fig. 1G). In the CPu, no significant changes in the levels of GSH were detected (Fig. 1H).

Inducible nitric oxide synthase (NOS) mRNA was also markedly increased in STZ‐diabetic mice after 4 weeks, whereas the mRNA encoding neuronal NOS showed a transient and relatively small increase (Fig. 1I). Together, these changes indicated the occurrence of oxidative stress in the brain associated with diabetes. This was further confirmed with the observation of higher levels of the oxidative stress marker 4‐HNE in the CPu of 4‐week STZ‐diabetic mice relative to nondiabetic controls (Fig. 1J), reflecting the presence of cellular damage.

Given that altered dopamine metabolism can contribute to increased vulnerability of nigrostriatal neurons by oxidative stress,^44^, ^45^ we investigated whether diabetes is accompanied by alterations in the content of dopamine and its metabolites in striatal axons. In STZ‐diabetic mice, we found decreased levels of dopamine, 3,4‐dihydroxyphenylacetic acid (DOPAC), and 3‐methoxytyramine (3‐MT) in the CPu relative to nondiabetic controls (Fig. 1K–M). Levels of noradrenaline, serotonin (5‐HT), or its metabolite, 5‐hydroxyindoleacetic acid (5‐HIAA), were similar in both groups (Supporting Information Fig. S5). Furthermore, DOPAC/dopamine and 3‐MT/dopamine ratios were decreased in STZ‐diabetic mice (Fig. 1N,O), indicating altered dopamine turnover. In *db/db* mice (blood glucose in *db/+* and *db/db* mice before experiments were 109 ± 3.1 and 515 ± 19.7 mg/dL, respectively), dopamine levels were similar, but DOPAC, 3‐MT, and their ratios to dopamine were decreased (Fig. 1P–T). These data indicate that striatal dopamine metabolism is affected in mouse models of both type 1 and type 2 diabetes.

**FIG. 2.**
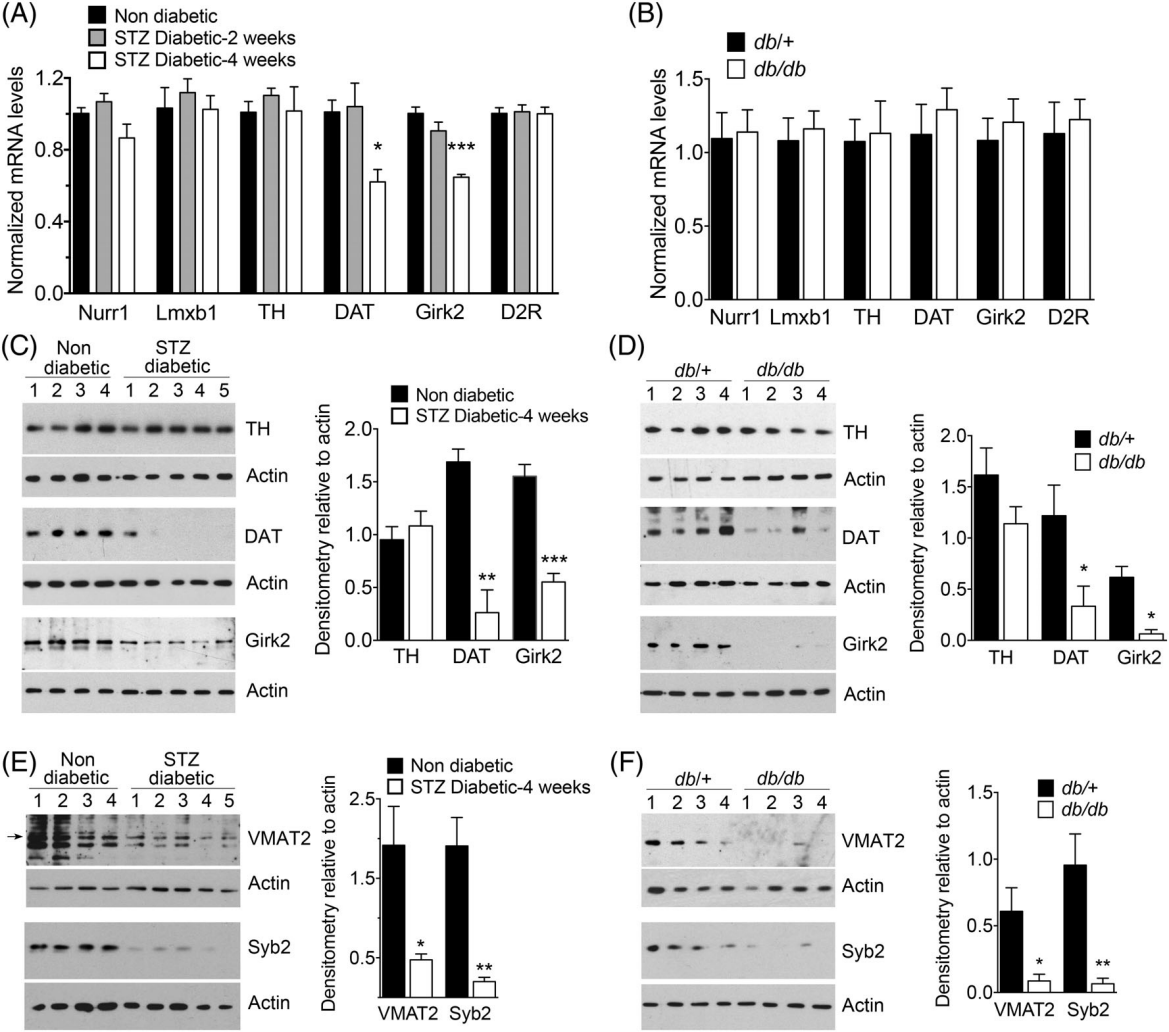
Diabetes decreases the striatal levels of axonal proteins in dopaminergic terminals. (**A,B**) Expression of mRNA encoding markers of dopamine neurons in the SN of nondiabetic or STZ‐diabetic mice (**A**; n = 6–7 per group) or of *db/*+ control and *db/db* diabetic mice (**B**; n = 8 per group). (**C–F**) Western blots with CPu lysates from nondiabetic or STZ‐diabetic (4 weeks) mice (**C,E**) or from *db/*+ control and *db/db* diabetic mice (**D,F**). Numbers on top of each lane indicate individual mice from which samples were obtained. The arrow in (**E**) indicates the band from which densitometric measurements were performed. Densitometric quantification of the bands is represented by histograms on the right side of each panel. *P < 0.05; **P < 0.01; ***P < 0.001 versus nondiabetic controls, one‐way ANOVA followed by Dunnett's post‐hoc test (**A**) or Student's *t* test (**C–F**). D2R, dopamine D2 autoreceptor

To investigate the possible damage to nigrostriatal neurons induced by diabetes, we determined the expression of dopaminergic neuron markers in the SN of STZ‐treated or *db/db* diabetic mice. We found no significant changes in levels of mRNAs encoding TH, the transcription factors nuclear receptor‐related 1 protein (Nurr1) and LIM homeobox transcription factor 1 beta (Lmxb1), or the dopamine D2 autoreceptors, indicating that diabetes per se did not affect the integrity of these neurons (Fig. 2A,B). Notably, levels of the mRNAs encoding DAT and the G‐protein‐activated inward rectifier potassium channel 2 (Girk2), two proteins expressed in dopaminergic neurons, were decreased in STZ‐treated mice (Fig. 2A). Western blots confirmed similar striatal levels of TH, suggesting the absence of a significant loss of dopaminergic fibers from nigrostriatal neurons, and decreased striatal levels of DAT and Girk2 in both models of diabetic mice, indicating the presence of specific changes in expression of key proteins regulating dopamine neurotransmission (Fig. 2C,D). Additional evidence for a possible dysfunction in striatal dopaminergic axons in both types of diabetic mice was the observation of decreased levels of the vesicular monoamine transporter 2 (VMAT2), and the vesicle‐associated protein, synaptobrevin‐2 (Syb2; Fig. 2E,F), without significant changes in expression of mRNAs encoding these proteins in the SN (Supporting Information Fig. S6).

**FIG. 3.**
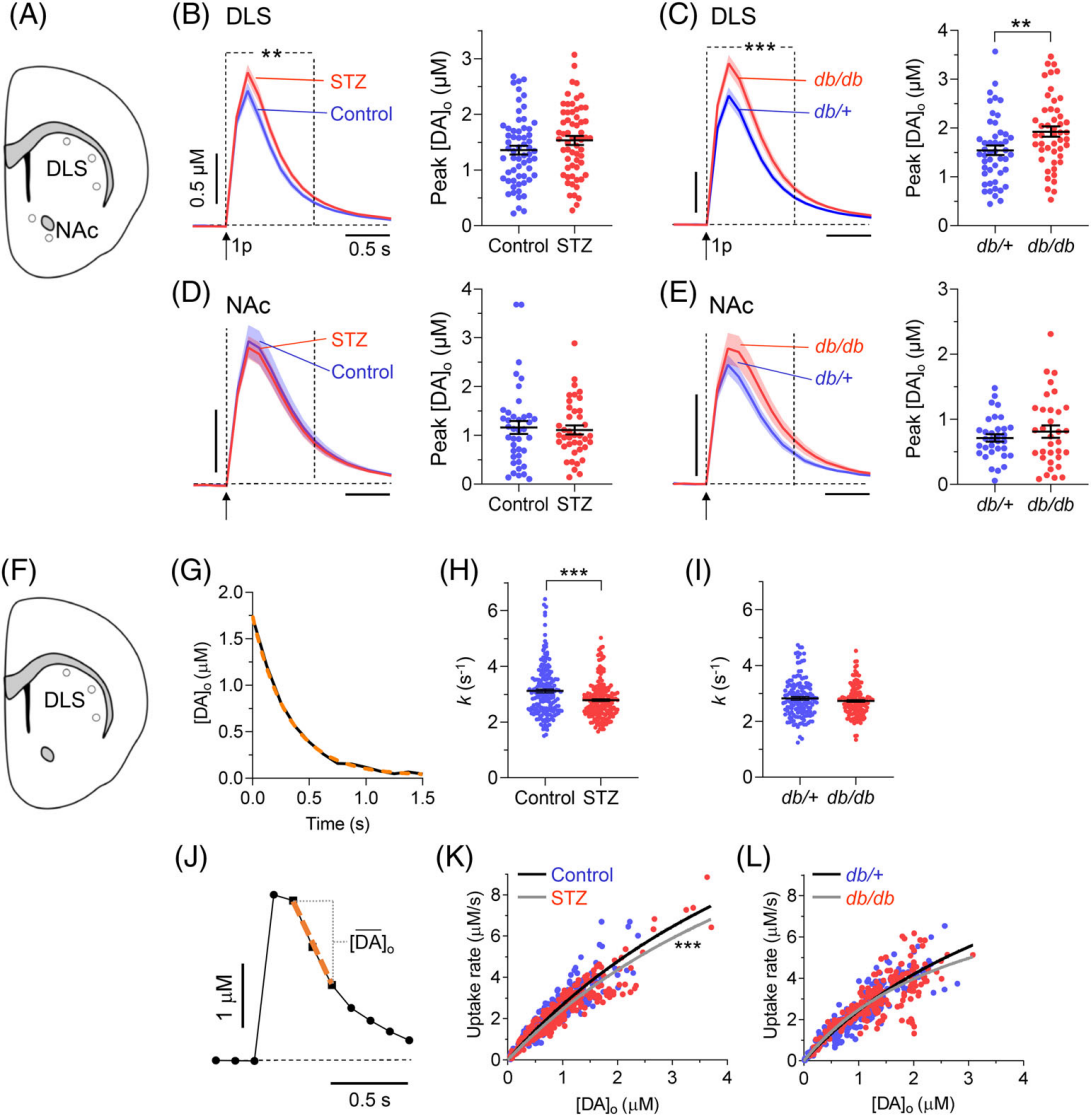
Dopamine release and uptake kinetics in the striatum. (**A**) Schematic depiction showing DLS and NAc regions sampled with FSCV. (**B,D**) Left: mean profiles of [DA]_o_ ± SEM evoked by a single electrical pulse in DLS (**B**; n = 61 recordings; *P* = 0.06, paired Student's *t* test) or NAc (**D**; n = 40 recordings) in vehicle‐treated nondiabetic controls (blue) or STZ‐treated 4‐week diabetic mice (red); ^**^ *P* < 0.01, two‐way ANOVA between dotted lines, treatment × time interaction, *F*_8,960_ = 2.682. Right: population data and mean ± SEM for peak [DA]_o_; ^*^ *P* < 0.05, paired Student's t test. (**C** and **E**) Left: mean profiles of [DA]_o_ ± SEM evoked by a single electrical pulse in DLS (**C**; n = 48 recordings) or NAc (**E**; n = 33 recordings) in *db/*+ nondiabetic controls (blue) and *db/db* diabetic mice (red); ^***^ *P* < 0.0001, two‐way ANOVA between dotted lines, treatment x time interaction, *F*_8,752_ = 4.422. Right, population data and mean ± SEM for peak [DA]_o_; ^**^ *P* < 0.01, paired Student's *t* test. Population data sets in (**B–E**) were checked for outliers with Grubb's test (α = 0.05). (**F**) Schematic depiction showing DLS regions assessed for uptake kinetics. (**G**) Representative fit (orange dashed) of a one‐phase exponential decay curve approximation to the falling phase of a typical extracellular dopamine profile (black; R^2^ = 0.99). (**H,I**) Population data and mean ± SEM for exponential decay constants (*k*) from approximation to one‐phase exponential decay fit calculated for each DA transient in STZ‐diabetic versus controls (**H**; n = 188; mean R^2^ > 0.98) or in *db/db* versus *db/+* (I; n = 142; mean R^2^ > 0.99). ^***^ *P*<0.0001, Student's *t* test. (**J**) A typical [DA]_o_ profile showing points of sampling the maximum [DA]_o_ decay rate (dashed orange line), and mean [DA]_o_ observed at this rate, for construction of a Michaelis‐Menten plot in (**K,L**). (**K,L**) Plot of maximum decay rate for each transient versus mean [DA]_o_ at that rate with Michaelis‐Menten curve fits for STZ‐diabetics (V_max_ = 20.1 ± 2.3 μM/s; R^2^ = 0.88) versus nondiabetic controls (V_max_ = 22.0 ± 2.6 μM/s; R^2^ = 0.83; K_m_ constrained to an equal value of 7.23 μM [K]; ^***^ *P* < 0.0001) and for *db/db* (V_max_ = 11.97 ± 1.23 μM/s; R^2^ = 0.67) versus *db/+* (V_max_ = 12.3 ± 1.3 μM/s; R^2^ = 0.83) mice (K_m_ constrained to an equal value of 3.9 μM for both genotypes; L). STZ‐diabetics versus controls, n = 10 pairs; *db/db* mice versus *db/+* controls, n = 8 pairs. [Color figure can be viewed at wileyonlinelibrary.com]

The decreased levels of proteins present in striatal axons led us to hypothesize that diabetes could alter dopamine neurotransmission in the CPu. To test this, we used FSCV to characterize dopamine release and uptake evoked in ex vivo slices of DLS and for comparison in NAc. In both STZ‐treated and *db/db* diabetic mice compared to their controls, we found a modest increase in the mean peak [DA]_o_ evoked by single electrical pulses in the DLS (to 113%, STZ; and 125%, *db/db*) and significant changes in the shape of mean extracellular dopamine transients (Fig. 3A–C). These effects were not observed in the NAc, where there was no significant change in mean peak or shape of [DA]_o_ profiles (Fig. 3D,E). To investigate whether in DLS there was an underlying decrease in dopamine uptake kinetics, we used two methods to analyze the falling phases of [DA]_o_ profiles—approximations to exponential decay and to a Michaelis‐Menten–like relationship—as described.^33^ In STZ‐diabetic, but not in *db/db*, mice, analyses of [DA]_o_ profiles revealed significantly lower values for mean uptake constant (Fig. 3F–I) and V_max_ (Fig. 3J–L) than in control mice.

**FIG. 4.**
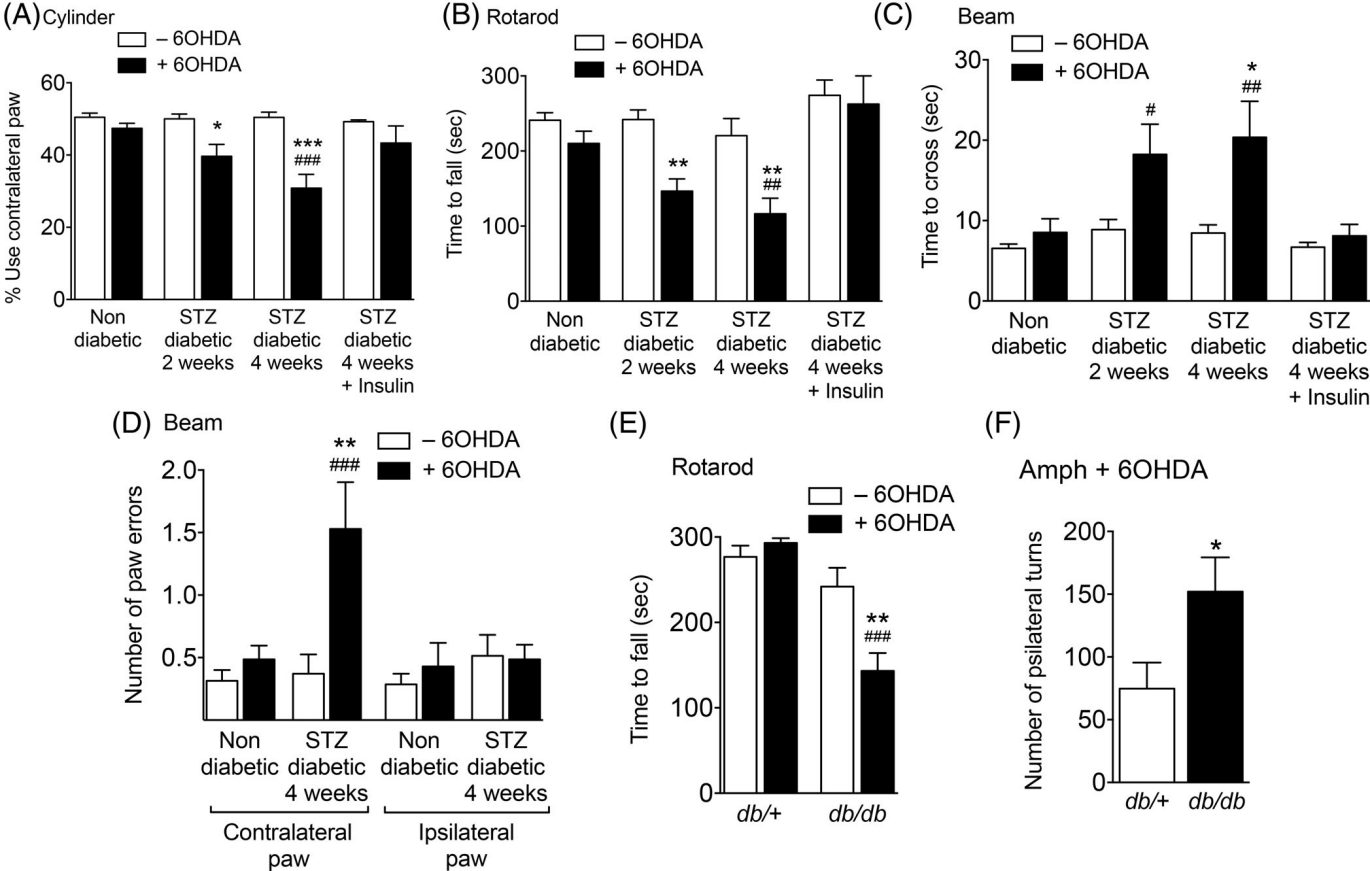
Motor performance before or after unilateral injection of a sub‐threshold dose of 6‐OHDA in the striatum. (**A–D**) Data from non‐diabetic or STZ‐diabetic mice (n = 8–14 per condition) evaluated on the cylinder (**A**), rotarod (**B**), or challenging traversal beam (**C,D**) tests. (**E,F**) Data from *db/*+ control or *db/db* diabetic mice (n = 8–11 per condition) on the rotarod test (**E**) or after induction of ipsilateral turns in response to administration of amphetamine (**F**). ^*^ *P* < 0.05; ^**^ *P* < 0.01; ^***^ *P* < 0.001 versus animals of the same group not injected with 6‐OHDA; ^#^ *P* < 0.05; ^##^ *P* < 0.01; ^###^ *P* < 0.001 versus 6‐OHDA‐injected nondiabetic mice; two‐way ANOVA followed by Bonferroni's post‐hoc test (**A–E**) or Student's *t* test (**F**).

The observed changes in diabetic mice did not translate into appreciable alterations in motor function (Fig. 4). Therefore, we investigated whether these changes could be associated with increased sensitization to nigrostriatal neurodegeneration. As expected, nondiabetic animals did not develop motor impairment after unilateral striatal microinjections of a subthreshold dose of 6‐OHDA (Fig. 4). In contrast, STZ‐treated animals showed decreased use of the paws contralateral to the injection side on the cylinder test (Fig. 4A), decreased latency to fall on the rotarod (Fig. 4B), and increased time to cross the beam on the challenging traversal test (Fig. 4C), the latter being correlated with an increased number of errors of the contralateral paws (Fig. 4D; Videos 1 and 2). Mice that were implanted with insulin pellets after STZ treatment did not develop motor impairments (Fig. 4A–C), indicating that these were not attributed to STZ toxicity. Furthermore, microinjection of 6‐OHDA into the striatum of *db/db* mice resulted in decreased latency to fall on the rotarod (Fig. 4E) and increased ipsilateral rotations after amphetamine injections relative to *db/+* controls (Fig. 4F). These results indicate that diabetes increases the susceptibility of mice to develop motor impairment attributable to dopaminergic damage.

**FIG. 5.**
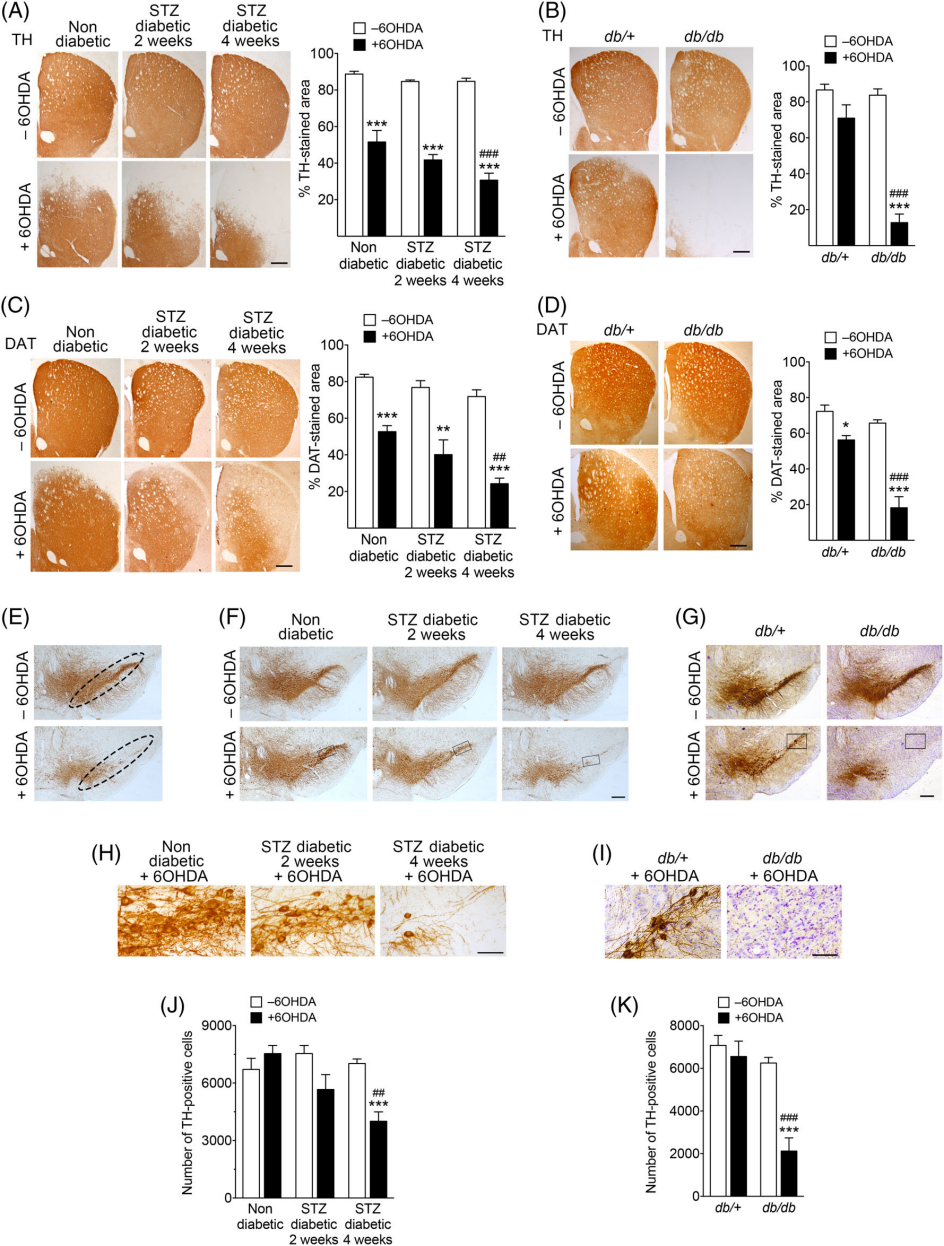
Increased nigrostriatal neurodegenerative damage in diabetic mice after 6‐OHDA administration. (**A–D**) Representative photomicrographs of coronal sections of the striatum of nondiabetic or STZ‐diabetic mice (**A,C**: n = 9–11 mice per group) or from *db/*+ control and *db/db* diabetic mice (**B,D**: n = 6–9 mice per group) immunostained for TH (**A,B**) or DAT (**C,D**). Sections of from the contralateral (–6‐OHDA) or ipsilateral (+6‐OHDA) side of 6‐OHDA injections are shown. Histograms represent the proportional stained area of TH or DAT immunoreactivity. (**E**) Representative images showing the region corresponding to the SNc used for stereological quantification of TH‐positive neurons. (**F,G**) Representative photomicrographs of coronal mesencephalic sections showing TH‐immunostained neurons in the SN (n = 5 mice per group). (**H,I**) High‐magnification images corresponding to the selected areas indicated by a rectangle in (**F**) and (**G**), respectively. (**J,K**) Quantification of the number of TH‐immunopositive neurons determined by stereology in the entire area corresponding to the SNc in each section. Scale bars represent 500 μm except in panels H and I (100 μm). In (**A**) to (**D**), at least four sections per animal were scored and averaged into a single point per animal. In (**F**) and (**G**), 10 sections per animal were scored to quantify the total number of TH‐positive cells in the SNc. ^*^ *P* = 0.05; ^**^ *P* < 0.01; ^***^ *P* < 0.001 relative to –6‐OHDA. ^##^ *P* < 0.01; ^###^ *P* < 0.001 versus +6‐OHDA‐injected nondiabetic mice (two‐way ANOVA followed by Bonferroni's post‐hoc test). [Color figure can be viewed at wileyonlinelibrary.com]

To confirm that motor impairment correlated with loss of nigrostriatal dopaminergic fibers, we performed immunohistochemical analyses. Unilateral microinjections of a subthreshold dose of 6‐OHDA induced a significantly greater loss of TH‐ and DAT‐immunopositive fibers in the striatum of STZ‐diabetic or *db/db* mice than in nondiabetic controls (Fig. 5A–D).

To determine whether loss of TH and DAT in the striatum of 6‐OHDA‐treated diabetic mice correlated with loss of mesencephalic dopamine neurons, we determined the number of TH‐immunoreactive neuronal cell bodies in the SNc by stereology (Fig. 5E). 6‐OHDA decreased the number of TH‐positive neurons in 4‐week STZ‐diabetic and *db/db* mice, whereas no significant neuronal loss was found in nondiabetic controls (Fig. 5F–K and Supporting Information Fig. S7), suggesting that nigral cell loss may be a component of motor deficits.

Furthermore, levels of striatal dopamine, DOPAC, and 3‐MT were lower in diabetic than in nondiabetic animals, both in the contra‐ and ipsilateral sides of 6‐OHDA injection (Supporting Information Fig. S8A–C). Such changes were not observed for other monoamines: Noradrenaline, 5‐HT, and 5‐HIAA were similar in both groups (Supporting Information Fig. S8D–F). Motor impairment was observed when dopamine loss, on average, exceeded 68% depletion (Supporting Information Fig. S9).

Together, these results indicate that the changes observed at the biochemical, molecular, and functional levels correlate with higher susceptibility of nigrostriatal dopaminergic neurons to neurodegeneration in diabetic mice than in nondiabetic controls.

## Discussion

STZ‐diabetic and *db/db* mice are two widely used models of type 1 and type 2 diabetes, respectively.^27^ STZ selectively destroys pancreatic islets, leading to lack of insulin and hyperglycemia, whereas in *db/db* mice diabetes follows obesity and insulin resistance generated by a recessive mutation in the leptin receptor gene. In agreement with previous studies,^46^, ^47^ both STZ‐treated and *db/db* mice exhibited increased vulnerability to nigrostriatal neurodegeneration. We now show that increased vulnerability in both types of diabetic animals is associated with decreased levels of proteins regulating dopamine neurotransmission and with altered stimulus‐dependent striatal dopamine release.

Whether PD is associated specifically with the more prevalent type 2 diabetes remains an open question.^48^ Both type 1 and type 2 diabetes have in common hyperglycemia and deficient insulin‐dependent signaling because of low insulin levels (type 1) or insulin resistance commonly associated with obesity (type 2). Both hyperglycemia and impaired insulin signaling are associated with PD.^14^, ^49^, ^50^ Regarding obesity, in high‐fat‐diet–induced rodent models showing increased susceptibility to nigrostriatal neurodegeneration, fasting hyperglycemia does not always reach levels of overt diabetes.^51^, ^52^, ^53^ Therefore, the relative contribution of hyperglycemia versus obesity remains unclear. Furthermore, some of these studies report decreased striatal dopamine release,^54^ whereas others show an increase.^55^ We found increased evoked [DA]_o_ in both STZ‐treated and *db/db* mice, consistent with the observed decrease in Girk2 and DAT. Notably, STZ‐treated mice are lean and *db/db* mice are obese, but both are diabetics. Therefore, our results indicate that obesity itself did not significantly contribute to striatal dysfunction in diabetic animals, and that hyperglycemia and decreased insulin‐dependent signaling are sufficient to generate dysfunctional dopamine neurotransmission. Importantly, a recent large‐scale study shows that underweight diabetic patients had higher risk for PD than overweight or obese patients.^56^ Therefore, whether obesity itself is a risk factor for PD remains an open question.^57^

We found that diabetes in lean STZ‐treated mice promotes oxidative stress‐related changes in the SN and CPu, in agreement with the well‐documented association between hyperglycemia and oxidative stress.^28^, ^58^, ^59^ Because of the relatively high‐energy demands that they require for function,^44^, ^60^ nigrostriatal dopaminergic neurons are particularly vulnerable to oxidative stress,^61^, ^62^, ^63^ which, in turn, is thought to play an important role in PD.^44^, ^64^, ^65^ Notably, 6‐OHDA used at a subthreshold dose did not produce detectable motor impairment in nondiabetic animals despite significant decreases in TH (∼42%) and DAT (∼36%) in the striatum, in line with previous studies.^66^, ^67^, ^68^, ^69^ In contrast, motor deficits were present 2 weeks after the onset of diabetes in STZ‐treated mice showing a loss of ∼50% (TH) and ∼47% (DAT). Given that neurotransmission by the remaining striatal dopaminergic axons in diabetic mice was altered, these experiments provide the basis for a mechanistic link between diabetes and impaired motor function related with increased vulnerability to neurodegeneration. Furthermore, motor deficits appeared to reflect dopamine neuron loss in the SN more closely than TH or dopamine loss in the striatum.

We found alterations in striatal proteins involved in the homeostasis of dopamine neurotransmission. Normally, released dopamine inhibits the activity of dopaminergic SN neurons and axons by negative feedback stimulation of dopamine D2 autoreceptors^70^ and striatal heteroreceptors,^71^ which can activate inwardly rectifying potassium Girk2 channels to hyperpolarize the membrane potential.^72^ Additionally, GABA_B_ receptors couple to Girks and inhibit striatal dopamine release directly.^73^ Girk2 (KCN6J) is relatively enriched in vulnerable SNc rather than spared ventral tegmental area dopamine neurons.^74^ If the reduction of Girk2 in diabetic mice impairs either of these feedback mechanisms, stimulus‐dependent dopamine release might be enhanced, particularly in the dorsal striatum, consistent with the higher evoked [DA]_o_ observed in the dorsal, but not ventral, striatum. Notably, decreased levels of Girk2 per se may contribute to neuronal vulnerability in diabetic animals even though its expression is not necessarily restricted to axons, consistent with the observation of nigrostriatal neurodegeneration in *weaver* mice carrying a mutation in the *Girk2* gene.^75^

Importantly, that overall levels of dopamine content in striatal samples are lower in STZ‐diabetic mice and not altered in *db/db* mice is not necessarily in conflict with the increased evoked [DA]_o_ detected by FSCV. Only 30% of dopamine‐containing vesicle clusters in striatal axons are associated with active secretory sites, and only ∼17% of VMAT2‐positive vesicles in those clusters release dopamine in response to depolarization.^76^, ^77^ Thus, most of the content of total striatal dopamine is likely contained within functionally silent VMAT2‐positive vesicles not immediately available for stimulus‐dependent release. Mobilization of the reserve vesicle pool provides a mechanism for increased dopamine release.^25^, ^78^, ^79^ Therefore, a redistribution in diabetic mice favoring mobilization of vesicles to releasable sites would be consistent with increased evoked [DA]_o_ even with reduced overall levels of VMAT2 and dopamine. Furthermore, a lack of components of the exocytosis machinery, such as synapsins, paradoxically increases dopamine release without altering the overall amount of dopamine.^80^

The decreased striatal DAT is consistent with reduced dopamine uptake and altered kinetic profile of [DA]_o_ transients, but reduced uptake was only observed in STZ‐diabetic mice. This suggests that *db/db* mice activate compensatory mechanisms to deliver DAT to the plasmalemmal membrane, possibly related to differences between both mouse models in insulin levels known to impact dopaminergic neurotransmission.^81^, ^82^, ^83^, ^84^ Consistent with our data, decreased DAT binding sites have been observed in diabetic patients with or without PD.^15^

Decreased VMAT2 levels may reflect decreased number or size of synaptic vesicles, in consonance with parallel low levels of Syb2, and suggest that the sequestration of axonal dopamine into vesicles is compromised in diabetic mice, favoring its accumulation in the cytosol, as indicated previously.^85^, ^86^, ^87^ Cytosolic dopamine contributes to neurodegeneration associated with oxidative stress.^85^, ^88^, ^89^, ^90^ Consistently, decreased expression of VMAT2 is sufficient to cause dopamine‐mediated toxicity and neurodegeneration of nigrostriatal neurons,^85^, ^91^ providing further mechanistic support for the association between diabetes and increased vulnerability to nigrostriatal neurodegeneration. Notably, our results agree with human studies showing decreased VMAT2 in early‐stage PD and with the observation that gain‐of‐function mutations are neuroprotective.^92^

Substantial axonal loss occurs in parkinsonian patients by the time of first diagnosis.^93^, ^94^ Our data show that diabetes in mice promotes striatal oxidative stress, dysfunctional dopamine neurotransmission associated with lower levels of key regulatory proteins, and increased susceptibility to damage of nigrostriatal neurons. Thus, our studies support the existence of an association between preexisting diabetes and PD at the molecular level and identify possible pathophysiological mechanisms affecting dopamine neurotransmission linking both diseases.

## Author Roles

(1) Research Project: A. Conception, B. Organization, C. Execution; (2) Statistical Analysis: A. Design, B. Execution, C. Review and Critique; (3) Manuscript: A. Writing of the First Draft, B. Review and Critique.

I.P.‐T.: 1C, 2B, 3B

S.A.: 1C, 2B, 3B

E.D.M.: 1B, 1C, 2B, 2C, 3B

R.A.: 1C, 2B, 3B

S.V.‐M.: 1C, 2B, 3B

N.N.T.: 1C, 2B, 3B

J.C.: 1B, 2C, 3B

S.J.C.: 1B, 2A, 2C, 3B

R.M.: 1A, 1B, 2A, 2C, 3B

M.V.: 1A, 1B, 2A, 2C, 3A, 3B

## Financial Disclosures

Nothing to report.

## Supporting information


**Supplementary Figure 1.** **Experimental design for mice treated with streptozotocin (STZ).** Animals were randomly assigned to three groups. In group 1, mice were killed either two or four weeks after the onset of diabetes to obtain samples from the striatum or substantia nigra. In group 2, 6‐OHDA was administered into the striatum two or four weeks after the onset of diabetes, and 10 days later motor tests were performed (a). After motor tests, mice were killed and their brains were processed for histology. A subgroup of four‐week diabetic animals was implanted with insulin pellets following STZ injections (b). The pellets were implanted immediately after the first determination of glucose levels in blood indicating hyperglycemia, approximately one week after the last STZ injection. In group 3, diabetes was let to proceed for four weeks and then fast‐scan cyclic voltammetry experiments were carried out during the following one to two weeks. Weeks are schematically represented as rectangles, and numbers are relative to the onset of diabetes. Weeks after the onset of diabetes are represented in gray.

**Supplementary Figure 2.** **Generation of diabetes by administration of streptozotocin (STZ). (A**) Blood glucose concentrations after fasting (4 hours). The dashed green line corresponds to glucose levels in animals that were implanted with insulin pellets immediately after the first hyperglycemic values were detected. In some data points of the vehicle and STZ + insulin groups the error bars are not visible due to their small size. **(B**) Evolution of body weight in mice treated with STZ or vehicle as indicated (mean + s.e.m.; n = 12 per group). Different groups of STZ‐treated mice were used in the study (see Supplementary Figure 1) yielding similar results.

**Supplementary Figure 3.** **Dissection of brain tissue.** Brains from mice fed *ad libitum* were rapidly removed and placed upside down in an acrylic rodent matrix (Cell Point Scientific, Gaithersburg, MD, USA) to generate coronal sections. **A)** To dissect a block of mesencephalic tissue containing the substantia nigra, a coronal cut was made at the level of the mammillary bodies (1), followed by another cut caudal to the edge of the brainstem (2). **B)** The resulting slice was placed on ice ventral side up. The mesencephalon was separated from the surrounding telencephalic tissue and diced into two pieces by a horizontal cut (3). Two parasagittal cuts (4) to discard a block of tissue at the midline were followed by two parallel 45° cuts above the dorsal side of the cerebral peduncle (5). A small tip of tissue remaining at the lateral side of the resulting block was also trimmed. **C)** To dissect the caudate putamen, a coronal slice was generated by making a first cut at the level of the rostral edge of the optic chiasm (6) and a second cut 2 mm rostral to the first one (7). **D)** The slice was placed on ice and the caudate putamen was dissected by making a diagonal cut with an end located approximately at the mid section of the lateral ventricle well above the anterior commisure to avoid the nucleus accumbens (8). The rest of the tissue was separated from the ventricle, corpus callosum and surrounding cortex. Drawings were modified from Paxinos, G. and Franklin, K.B.J., The mouse brain in stereotaxic coordinates, Academic Press, San Diego, 2001. Abbreviations: CC, Corpus callosum; CPu, Caudate putamen; MB, Mammillary body; NAc, Nucleus accumbens; SNc, Substantia nigra pars compacta. Scale bar, 1 mm.

**Supplementary Figure 4.** **Determination of the stability of *Gapdh* expression as a reference in RT‐qPCR experiments.** GeNorm analyses using mRNA from mesencephalic samples containing the substantia nigra obtained from control nondiabetic and STZ‐treated diabetic mice **(A)** or from control *db/+* and diabetic *db/db* mice **(B)**. Sdha, succinate dehydrogenase A subunit; B2M, beta‐2 microglobulin; Hprt, hypoxanthine‐guanine phosphoribosyltransferase; Gapdh, glyceraldehyde 3‐phosphate dehydrogenase. Note that the M stability value for *Gapdh* is well below 0.5 considered acceptable for homogeneous samples (n = 16 per group).

**Supplementary Figure 5.** Levels of noradrenaline **(A)**, serotonin (5‐HT) **(B)** and its metabolite 5‐HIAA **(C)** determined by HPLC in homogenates of the caudate putamen. Data correspond to non‐diabetic mice or to mice that had been diabetic for 2 or 4 weeks (2W or 4W, respectively) after streptozotocin administration (n = 6 per group).

**Supplementary Figure 6.** Diabetes does not affect the expression of genes encoding the presynaptic proteins VMAT2 and synaptobrevin‐2 (Syb2). Shown are the levels of expression of mRNA detected by RT‐qPCR in the mesencephalic substantia nigra of STZ‐treated or *db/db* diabetic mice and their respective controls(**A**, n = 6‐7 per group; **B**, n = 8 per group).

**Supplementary Figure 7.** **Increased cell loss in the substantia nigra of diabetic mice after 6‐ OHDA administration. A** and **B**) Photomicrographs of coronal mesencephalic sections showing TH‐immunostained neurons counterstained with Nissl stain in the substantia nigra of control nondiabetic or four‐week STZ‐diabetic mice treated with a subthreshold dose of 6‐OHDA. The images correspond approximately to the areas indicated with a rectangle in Figure 5F of the main text. Scale bar, 60 μm. **C** and **D)** Quantification of the number of Nissl‐stained cells determined by stereology in STZ‐treated mice and their controls **(C)** or in *db/db* mice and their *db/+* controls **(D)**. Representative images for *db/db* mice used for quantification correspond to those shown in Figure 5I. Ten sections per animal were scored (n=3 mice per group). **P<0.0 relative to –6‐OHDA. #*P*<0.05, ##*P*<0.01 versus +6‐OHDA‐injected non‐diabetic controls (two‐way ANOVA followed by Bonferroni post hoc test). The calculated decrease in TH‐positive neurons (see Figure 5 in the main text) was not significantly different to that calculated with the Nissl staining (3011 ± 590 versus 3680 ± 512, for four‐week STZ‐diabetic mice; and 4128 ± 605 versus 4018 ± 928 for *db/db* mice, respectively), indicating that cell loss was specific to dopaminergic neurons.

**Supplementary Figure 8.** **A‐F**) Levels of dopamine **(A)**, DOPAC **(B)**, 3‐MT **(C)** noradrenaline **(D)** serotonin **(E)** and 5‐HIAA **(F)** in the caudate putamen of mice treated unilaterally with microinjections of 6‐OHDA. Data from the ipsilateral and contralateral striata are shown. ND, non‐diabetic mice (black columns); STZ‐D, STZ‐treated diabetic mice (white columns); 2w or 4w, mice that had been diabetic for 2 or 4 weeks after STZ injections. *P < 0.05; **P < 0.01; ***P < 0.001 versus non‐diabetic controls; one‐way ANOVA followed by Dunnett's post hoc test (n=11‐15 per group).

**Supplementary Figure 9.** Dopamine depletion detected in the striatum of 6‐OHDA treated non‐diabetic (ND) or STZ‐treated diabetic mice (STZ‐D). Data from the ipsilateral and contralateral striata relative to the 6‐OHDA injection side are shown. Data (mean + sem) were calculated from those presented in Supplementary Figure 8A as a percentage of the mean value corresponding to the non‐diabetic contralateral side. The doted red line denotes the threshold value below which motor impairment was not observed, and corresponds to the value obtained in the ipsilateral side of two‐week STZdiabetic mice (68.56 + 3.8%). 2w or 4w, mice that had been diabetic for 2 or 4 weeks after STZ injections.

**Supplementary Table 1.** Antibodies used in western blots or immunohistochemistry.

**Video 1.** Movie showing a representative nondiabetic mouse previously treated with a subthreshold dose of 6‐hydroxydopamine unilaterally into the striatum, performing the challenging traversal beam test.

**Video 2.** Movie showing a representative STZ‐treated diabetic mouse previously injected with a subthreshold dose of 6‐hydroxydopamine unilaterally into the striatum 4 weeks after the onset of hyperglycemia, performing the challenging traversal beam test. Note that the mouse takes longer to cross than a nondiabetic control, and misses the grid when stepping with the paws on the observer side, contralateral to the 6‐hydroxydopamine injection side.
