# Supplementary material for: Diabetes Causes Dysfunctional Dopamine Neurotransmission Favoring Nigrostriatal Degeneration in Mice
Source: Mov Disord. 2020 Jul 15;35(9):1636–48. doi: 10.1002/mds.28124 (PMC7818508; doi:10.1002/mds.28124)
Supplement: Supplementary file 1 — Supplementary Figure 1. Experimental design for mice treated with streptozotocin (STZ). Animals were randomly assigned to three groups. In group 1, mice were killed either two or four weeks after the onset of diabetes to obtain samples from the striatum or substantia nigra. In group 2, 6‐OHDA was administered into the striatum two or four weeks after the onset of diabetes, and 10 days later motor tests were performed (a). After motor tests, mice were killed and their brains were processed for histology. A subgroup of four‐week diabetic animals was implanted with insulin pellets following STZ injections (b). The pellets were implanted immediately after the first determination of glucose levels in blood indicating hyperglycemia, approximately one week after the last STZ injection. In group 3, diabetes was let to proceed for four weeks and then fast‐scan cyclic voltammetry experiments were carried out during the following one to two weeks. Weeks are schematically represented as rectangles, and numbers are relative to the onset of diabetes. Weeks after the onset of diabetes are represented in gray. [file MDS-35-1636-s001.pdf]

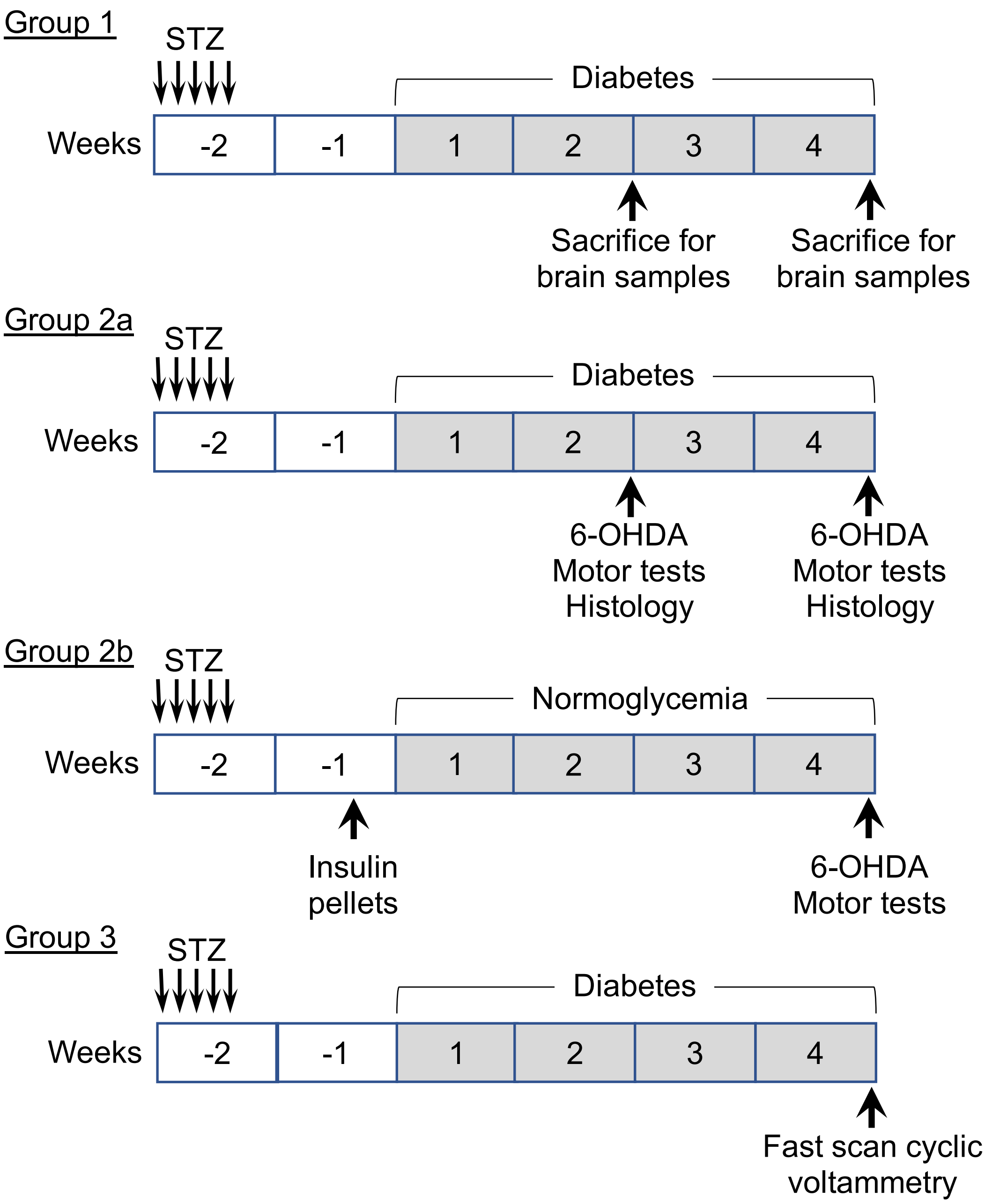

Supplementary Figure 1. Experimental design for mice treated with streptozotocin (STZ). Animals were randomly assigned to three groups. In group 1, mice were killed either two or four weeks after the onset of diabetes to obtain samples from the striatum or substantia nigra. In group 2, 6-OHDA was administered into the striatum two or four weeks after the onset of diabetes, and 10 days later motor tests were performed (a). After motor tests, mice were killed and their brains were processed for histology. A subgroup of four-week diabetic animals was implanted with insulin pellets following STZ injections (b). The pellets were implanted immediately after the first determination of glucose levels in blood indicating hyperglycemia, approximately one week after the last STZ injection. In group 3, diabetes was let to proceed for four weeks and then fast-scan cyclic voltammetry experiments were carried out during the following one to two weeks. Weeks are schematically represented as rectangles, and numbers are relative to the onset of diabetes. Weeks after the onset of diabetes are represented in gray.
