# Supplementary material for: Diabetes Causes Dysfunctional Dopamine Neurotransmission Favoring Nigrostriatal Degeneration in Mice
Source: Mov Disord. 2020 Jul 15;35(9):1636–48. doi: 10.1002/mds.28124 (PMC7818508; doi:10.1002/mds.28124)
Supplement: Supplementary file 2 — Supplementary Figure 2. Generation of diabetes by administration of streptozotocin (STZ). (A) Blood glucose concentrations after fasting (4 hours). The dashed green line corresponds to glucose levels in animals that were implanted with insulin pellets immediately after the first hyperglycemic values were detected. In some data points of the vehicle and STZ + insulin groups the error bars are not visible due to their small size. (B) Evolution of body weight in mice treated with STZ or vehicle as indicated (mean + s.e.m.; n = 12 per group). Different groups of STZ‐treated mice were used in the study (see Supplementary Figure 1) yielding similar results. [file MDS-35-1636-s004.pdf]

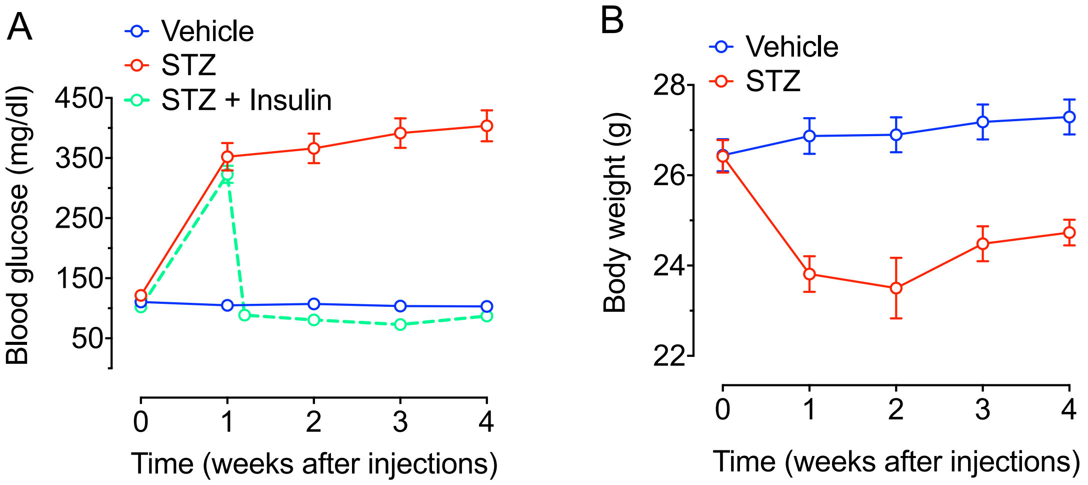

Supplementary Figure 2. Generation of diabetes by administration of streptozotocin (STZ). (A) Blood glucose concentrations after fasting (4 hours). The dashed green line corresponds to glucose levels in animals that were implanted with insulin pellets immediately after the first hyperglycemic values were detected. In some data points of the vehicle and STZ + insulin groups the error bars are not visible due to their small size. **(B)** Evolution of body weight in mice treated with STZ or vehicle as indicated (mean ± s.e.m.; n = 12 per group). Different groups of STZ-treated mice were used in the study (see Supplementary Figure 1) yielding similar results.
