# Supplementary material for: Diabetes Causes Dysfunctional Dopamine Neurotransmission Favoring Nigrostriatal Degeneration in Mice
Source: Mov Disord. 2020 Jul 15;35(9):1636–48. doi: 10.1002/mds.28124 (PMC7818508; doi:10.1002/mds.28124)
Supplement: Supplementary file 3 — Supplementary Figure 3. Dissection of brain tissue. Brains from mice fed ad libitum were rapidly removed and placed upside down in an acrylic rodent matrix (Cell Point Scientific, Gaithersburg, MD, USA) to generate coronal sections. A) To dissect a block of mesencephalic tissue containing the substantia nigra, a coronal cut was made at the level of the mammillary bodies (1), followed by another cut caudal to the edge of the brainstem (2). B) The resulting slice was placed on ice ventral side up. The mesencephalon was separated from the surrounding telencephalic tissue and diced into two pieces by a horizontal cut (3). Two parasagittal cuts (4) to discard a block of tissue at the midline were followed by two parallel 45° cuts above the dorsal side of the cerebral peduncle (5). A small tip of tissue remaining at the lateral side of the resulting block was also trimmed. C) To dissect the caudate putamen, a coronal slice was generated by making a first cut at the level of the rostral edge of the optic chiasm (6) and a second cut 2 mm rostral to the first one (7). D) The slice was placed on ice and the caudate putamen was dissected by making a diagonal cut with an end located approximately at the mid section of the lateral ventricle well above the anterior commisure to avoid the nucleus accumbens (8). The rest of the tissue was separated from the ventricle, corpus callosum and surrounding cortex. Drawings were modified from Paxinos, G. and Franklin, K.B.J., The mouse brain in stereotaxic coordinates, Academic Press, San Diego, 2001. Abbreviations: CC, Corpus callosum; CPu, Caudate putamen; MB, Mammillary body; NAc, Nucleus accumbens; SNc, Substantia nigra pars compacta. Scale bar, 1 mm. [file MDS-35-1636-s006.pdf]

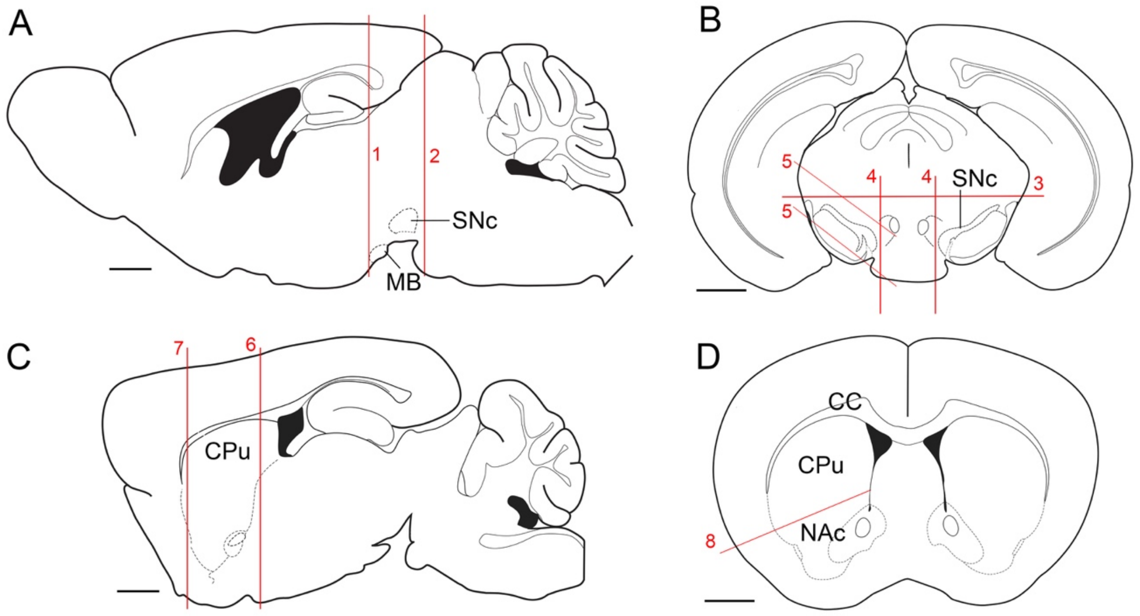

Supplementary Figure 3. Dissection of brain tissue. Brains from mice fed *ad libitum* were rapidly removed and placed upside down in an acrylic rodent matrix (Cell Point Scientific, Gaithersburg, MD, USA) to generate coronal sections. **A)** To dissect a block of mesencephalic tissue containing the substantia nigra, a coronal cut was made at the level of the mammillary bodies (1), followed by another cut caudal to the edge of the brainstem (2). **B)** The resulting slice was placed on ice ventral side up. The mesencephalon was separated from the surrounding telencephalic tissue and diced into two pieces by a horizontal cut (3). Two parasagittal cuts (4) to discard a block of tissue at the midline were followed by two parallel 45° cuts above the dorsal side of the cerebral peduncle (5). A small tip of tissue remaining at the lateral side of the resulting block was also trimmed. **C)** To dissect the caudate putamen, a coronal slice was generated by making a first cut at the level of the rostral edge of the optic chiasm (6) and a second cut 2 mm rostral to the first one (7). **D)** The slice was placed on ice and the caudate putamen was dissected by making a diagonal cut with an end located approximately at the mid section of the lateral ventricle well above the anterior commisure to avoid the nucleus accumbens (8). The rest of the tissue was separated from the ventricle, corpus callosum and surrounding cortex. Drawings were modified from Paxinos, G. and Franklin, K.B.J., The mouse brain in stereotaxic coordinates, Academic Press, San Diego, 2001. Abbreviations: CC, Corpus callosum; CPu, Caudate putamen; MB, Mammillary body; NAc, Nucleus accumbens; SNc, Substantia nigra pars compacta. Scale bar, 1 mm.
