# Supplementary material for: Diabetes Causes Dysfunctional Dopamine Neurotransmission Favoring Nigrostriatal Degeneration in Mice
Source: Mov Disord. 2020 Jul 15;35(9):1636–48. doi: 10.1002/mds.28124 (PMC7818508; doi:10.1002/mds.28124)
Supplement: Supplementary file 4 — Supplementary Figure 4. Determination of the stability of Gapdh expression as a reference in RT‐qPCR experiments. GeNorm analyses using mRNA from mesencephalic samples containing the substantia nigra obtained from control nondiabetic and STZ‐treated diabetic mice (A) or from control db/+ and diabetic db/db mice (B). Sdha, succinate dehydrogenase A subunit; B2M, beta‐2 microglobulin; Hprt, hypoxanthine‐guanine phosphoribosyltransferase; Gapdh, glyceraldehyde 3‐phosphate dehydrogenase. Note that the M stability value for Gapdh is well below 0.5 considered acceptable for homogeneous samples (n = 16 per group). [file MDS-35-1636-s007.pdf]

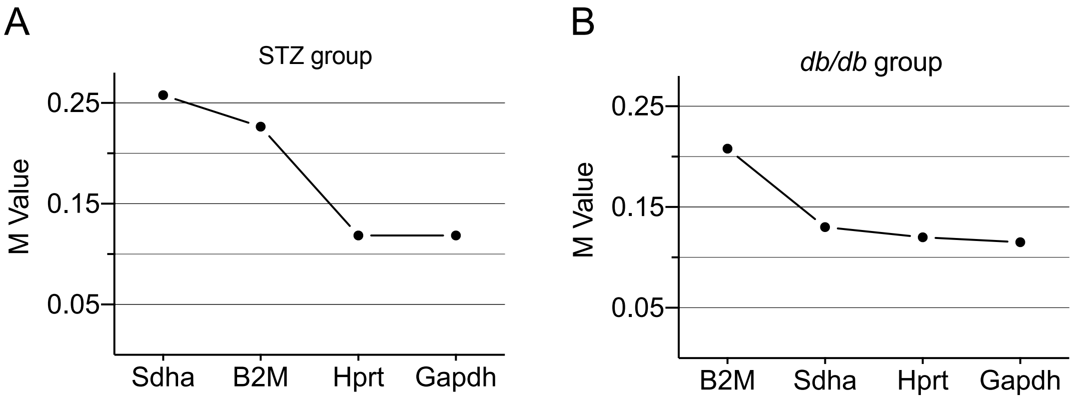

Supplementary Figure 4. Determination of the stability of *Gapdh* expression as a reference in RT-qPCR experiments. GeNorm analyses using mRNA from mesencephalic samples containing the substantia nigra obtained from control non-diabetic and STZ-treated diabetic mice **(A)** or from control *db*/+ and diabetic *db*/*db* mice **(B)**. Sdha, succinate dehydrogenase A subunit; B2M, beta-2 microglobulin; Hprt, hypoxanthine-guanine phosphoribosyltransferase; Gapdh, glyceraldehyde 3-phosphate dehydrogenase. Note that the M stability value for *Gapdh* is well below 0.5 considered acceptable for homogeneous samples (n = 16 per group).
