# Supplementary material for: Diabetes Causes Dysfunctional Dopamine Neurotransmission Favoring Nigrostriatal Degeneration in Mice
Source: Mov Disord. 2020 Jul 15;35(9):1636–48. doi: 10.1002/mds.28124 (PMC7818508; doi:10.1002/mds.28124)
Supplement: Supplementary file 5 — Supplementary Figure 5. Levels of noradrenaline (A), serotonin (5‐HT) (B) and its metabolite 5‐HIAA (C) determined by HPLC in homogenates of the caudate putamen. Data correspond to non‐diabetic mice or to mice that had been diabetic for 2 or 4 weeks (2W or 4W, respectively) after streptozotocin administration (n = 6 per group). [file MDS-35-1636-s008.pdf]

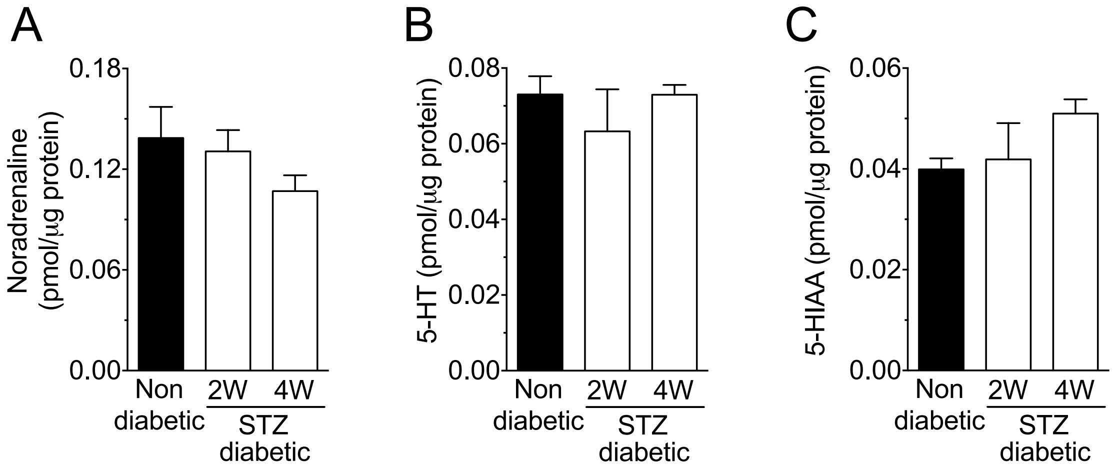

Supplementary Figure 5. Levels of noradrenaline **(A)**, serotonin (5-HT) **(B)** and its metabolite 5-HIAA **(C)** determined by HPLC in homogenates of the caudate putamen. Data correspond to non-diabetic mice or to mice that had been diabetic for 2 or 4 weeks (2W or 4W, respectively) after streptozotocin administration (n = 6 per group).
