# Supplementary material for: Diabetes Causes Dysfunctional Dopamine Neurotransmission Favoring Nigrostriatal Degeneration in Mice
Source: Mov Disord. 2020 Jul 15;35(9):1636–48. doi: 10.1002/mds.28124 (PMC7818508; doi:10.1002/mds.28124)
Supplement: Supplementary file 6 — Supplementary Figure 6. Diabetes does not affect the expression of genes encoding the presynaptic proteins VMAT2 and synaptobrevin‐2 (Syb2). Shown are the levels of expression of mRNA detected by RT‐qPCR in the mesencephalic substantia nigra of STZ‐treated or db/db diabetic mice and their respective controls(A, n = 6‐7 per group; B, n = 8 per group). [file MDS-35-1636-s009.pdf]

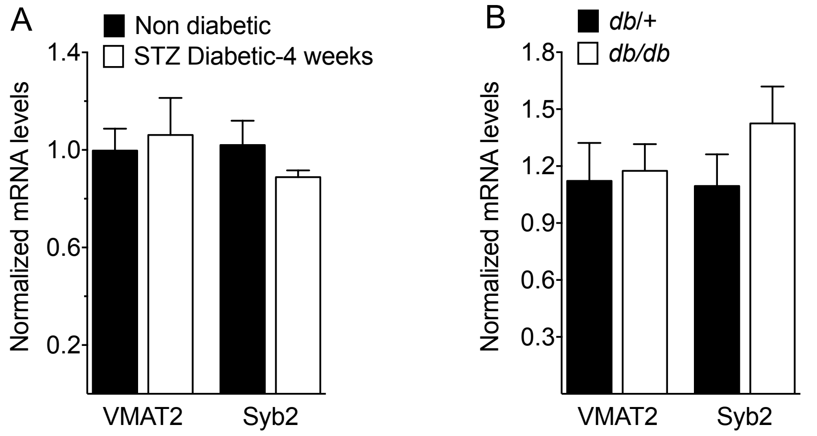

Supplementary Figure 6. Diabetes does not affect the expression of genes encoding the presynaptic proteins VMAT2 and synaptobrevin-2 (Syb2). Shown are the levels of expression of mRNA detected by RT-qPCR in the mesencephalic substantia nigra of STZ-treated or *db*/*db* diabetic mice and their respective controls(**A**, n = 6-7 per group; **B**, n = 8 per group).
