# Supplementary material for: Diabetes Causes Dysfunctional Dopamine Neurotransmission Favoring Nigrostriatal Degeneration in Mice
Source: Mov Disord. 2020 Jul 15;35(9):1636–48. doi: 10.1002/mds.28124 (PMC7818508; doi:10.1002/mds.28124)
Supplement: Supplementary file 7 — Supplementary Figure 7. Increased cell loss in the substantia nigra of diabetic mice after 6‐ OHDA administration. A and B) Photomicrographs of coronal mesencephalic sections showing TH‐immunostained neurons counterstained with Nissl stain in the substantia nigra of control nondiabetic or four‐week STZ‐diabetic mice treated with a subthreshold dose of 6‐OHDA. The images correspond approximately to the areas indicated with a rectangle in Figure 5F of the main text. Scale bar, 60 μm. C and D) Quantification of the number of Nissl‐stained cells determined by stereology in STZ‐treated mice and their controls (C) or in db/db mice and their db/+ controls (D). Representative images for db/db mice used for quantification correspond to those shown in Figure 5I. Ten sections per animal were scored (n=3 mice per group). **P<0.0 relative to –6‐OHDA. #P<0.05, ##P<0.01 versus +6‐OHDA‐injected non‐diabetic controls (two‐way ANOVA followed by Bonferroni post hoc test). The calculated decrease in TH‐positive neurons (see Figure 5 in the main text) was not significantly different to that calculated with the Nissl staining (3011 ± 590 versus 3680 ± 512, for four‐week STZ‐diabetic mice; and 4128 ± 605 versus 4018 ± 928 for db/db mice, respectively), indicating that cell loss was specific to dopaminergic neurons. [file MDS-35-1636-s010.pdf]

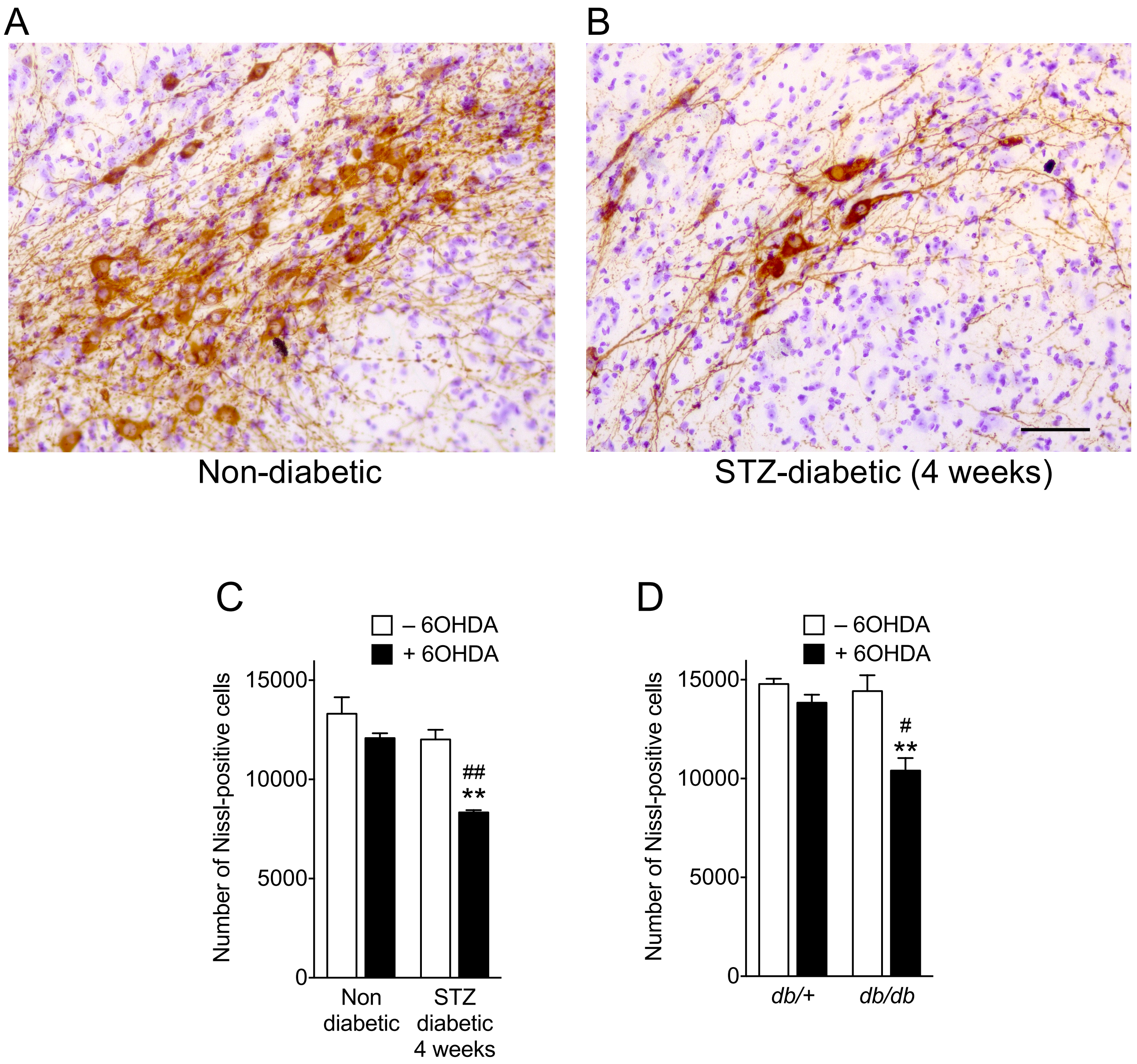

Supplementary Figure 7. Increased cell loss in the substantia nigra of diabetic mice after 6-OHDA administration. A and **B**) Photomicrographs of coronal mesencephalic sections showing TH-immunostained neurons counterstained with Nissl stain in the substantia nigra of control non-diabetic or four-week STZ-diabetic mice treated with a subthreshold dose of 6-OHDA. The images correspond approximately to the areas indicated with a rectangle in Figure 5F of the main text. Scale bar, 60 µm. **C** and **D**) Quantification of the number of Nissl-stained cells determined by stereology in STZ-treated mice and their controls (**C**) or in *db*/*db* mice and their *db*/+ controls (**D**). Representative images for *db*/*db* mice used for quantification correspond to those shown in Figure 5I. Ten sections per animal were scored (n=3 mice per group). $^{**}P<0.0$ relative to –6-OHDA. $^{\#}P<0.05$, $^{\#\#}P<0.01$ versus +6-OHDA-injected non-diabetic controls (two-way ANOVA followed by Bonferroni post hoc test). The calculated decrease in TH-positive neurons (see Figure 5 in the main text) was not significantly different to that calculated with the Nissl staining (3011 ± 590 versus 3680 ± 512, for four-week STZ-diabetic mice; and 4128 ± 605 versus 4018 ± 928 for *db*/*db* mice, respectively), indicating that cell loss was specific to dopaminergic neurons.
