# Supplementary material for: Diabetes Causes Dysfunctional Dopamine Neurotransmission Favoring Nigrostriatal Degeneration in Mice
Source: Mov Disord. 2020 Jul 15;35(9):1636–48. doi: 10.1002/mds.28124 (PMC7818508; doi:10.1002/mds.28124)
Supplement: Supplementary file 8 — Supplementary Figure 8. A‐F) Levels of dopamine (A), DOPAC (B), 3‐MT (C) noradrenaline (D) serotonin (E) and 5‐HIAA (F) in the caudate putamen of mice treated unilaterally with microinjections of 6‐OHDA. Data from the ipsilateral and contralateral striata are shown. ND, non‐diabetic mice (black columns); STZ‐D, STZ‐treated diabetic mice (white columns); 2w or 4w, mice that had been diabetic for 2 or 4 weeks after STZ injections. *P < 0.05; **P < 0.01; ***P < 0.001 versus non‐diabetic controls; one‐way ANOVA followed by Dunnett's post hoc test (n=11‐15 per group). [file MDS-35-1636-s011.pdf]

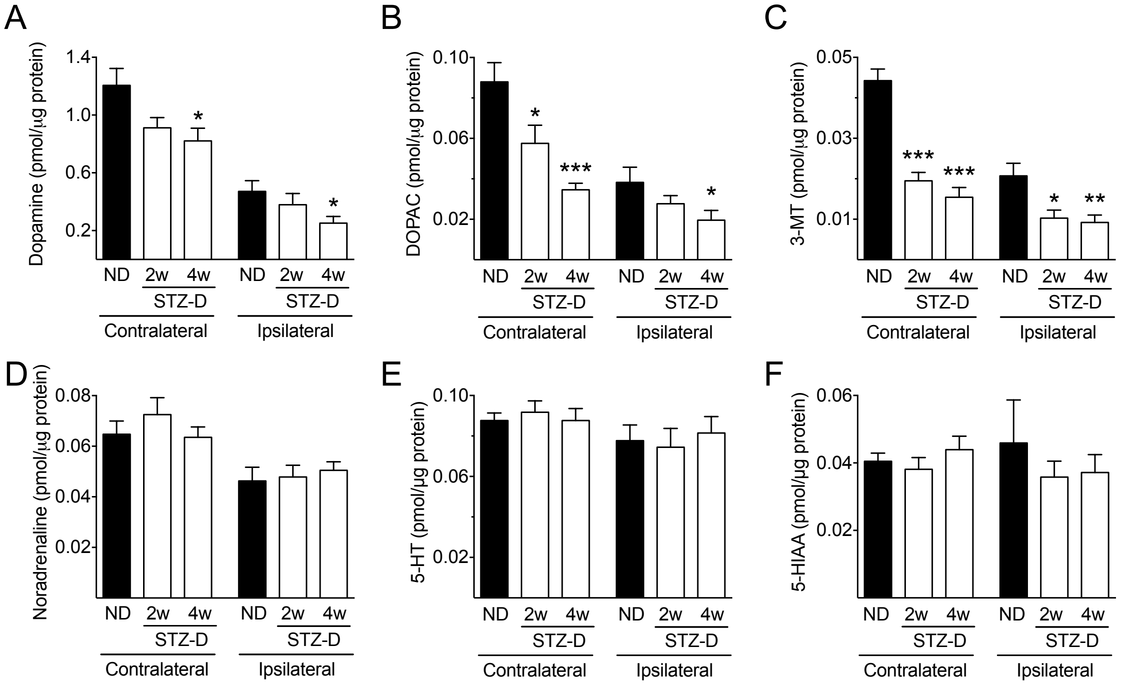

Supplementary Figure 8. A-F) Levels of dopamine (**A**), DOPAC (**B**), 3-MT (**C**) noradrenaline (**D**) serotonin (**E**) and 5-HIAA (**F**) in the caudate putamen of mice treated unilaterally with microinjections of 6-OHDA. Data from the ipsilateral and contralateral striata are shown. ND, non-diabetic mice (black columns); STZ-D, STZ-treated diabetic mice (white columns); 2w or 4w, mice that had been diabetic for 2 or 4 weeks after STZ injections. *$P < 0.05$; **$P < 0.01$; ***$P < 0.001$ versus non-diabetic controls; one-way ANOVA followed by Dunnett's post hoc test (n=11-15 per group).
