# Supplementary material for: Diabetes Causes Dysfunctional Dopamine Neurotransmission Favoring Nigrostriatal Degeneration in Mice
Source: Mov Disord. 2020 Jul 15;35(9):1636–48. doi: 10.1002/mds.28124 (PMC7818508; doi:10.1002/mds.28124)
Supplement: Supplementary file 9 — Supplementary Figure 9. Dopamine depletion detected in the striatum of 6‐OHDA treated non‐diabetic (ND) or STZ‐treated diabetic mice (STZ‐D). Data from the ipsilateral and contralateral striata relative to the 6‐OHDA injection side are shown. Data (mean + sem) were calculated from those presented in Supplementary Figure 8A as a percentage of the mean value corresponding to the non‐diabetic contralateral side. The doted red line denotes the threshold value below which motor impairment was not observed, and corresponds to the value obtained in the ipsilateral side of two‐week STZdiabetic mice (68.56 + 3.8%). 2w or 4w, mice that had been diabetic for 2 or 4 weeks after STZ injections. [file MDS-35-1636-s012.pdf]

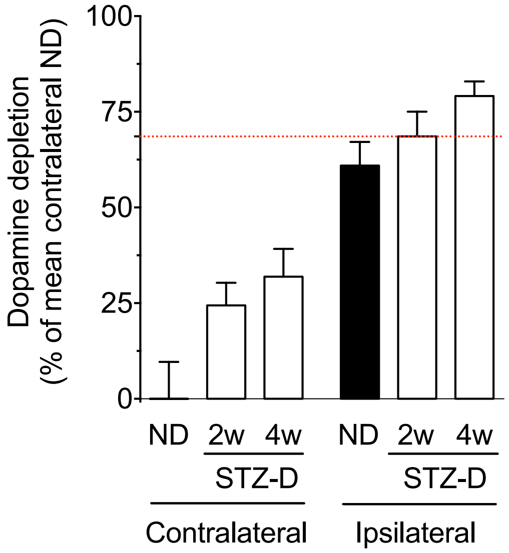

Supplementary Figure 9. Dopamine depletion detected in the striatum of 6-OHDA treated non-diabetic (ND) or STZ-treated diabetic mice (STZ-D). Data from the ipsilateral and contralateral striata relative to the 6-OHDA injection side are shown. Data (mean $\pm$ sem) were calculated from those presented in Supplementary Figure 8A as a percentage of the mean value corresponding to the non-diabetic contralateral side. The doted red line denotes the threshold value below which motor impairment was not observed, and corresponds to the value obtained in the ipsilateral side of two-week STZ-diabetic mice (68.56 $\pm$ 3.8%). 2w or 4w, mice that had been diabetic for 2 or 4 weeks after STZ injections.
