# Supplementary material for: Diabetes Causes Dysfunctional Dopamine Neurotransmission Favoring Nigrostriatal Degeneration in Mice
Source: Mov Disord. 2020 Jul 15;35(9):1636–48. doi: 10.1002/mds.28124 (PMC7818508; doi:10.1002/mds.28124)
Supplement: Supplementary file 10 — Supplementary Table 1. Antibodies used in western blots or immunohistochemistry. [file MDS-35-1636-s002.pdf]

Supplementary Table 1. Antibodies used in western blots or immunohistochemistry.

**Antibody**	**Source**	**Reference**	**Dilution**
Catalase	Santa Cruz Biotechnology	Sc-271803	1:1000
Superoxide dismutase 2	Abcam	ab13533	1:5000
VMAT2	Merck	AB1598P	1:1000
Synaptobrevin 2	Synaptic Systems	104 202	1:500
Tyrosine hydroxilase	Chemicon International	ab152	1:1000
Dopamine transporter	Abcam	ab5990	1:1000
Girk2	Abcam	ab65096	1:500
β-Actin	Sigma-Aldrich	AC-15	1:10000
